# Non-linear association between neutrophil-to-lymphocyte ratio and 90-day mortality in patients with pneumonia receiving glucocorticoids alone or in combination with other immunosuppressants: A retrospective cohort study

**DOI:** 10.1371/journal.pone.0329616

**Published:** 2025-08-18

**Authors:** Caizhen Chen, Xiuguo Zhang, Baoli Li, Qian Geng

**Affiliations:** Department of Nursing, Third Hospital of Hebei Medical University, Shijiazhuang, Hebei, China; University of Colombo Faculty of Medicine, SRI LANKA

## Abstract

**Objective:**

The relationship between neutrophil-to-lymphocyte ratio (NLR) and 90-day mortality in patients with pneumonia receiving glucocorticoids alone or in combination with other immunosuppressants has not been fully verified. We aimed to explore the influence of NLR on 90-day mortality in this specific population.

**Methods:**

This study utilized the data set from the Dryad database, involving 696 participants diagnosed with pneumonia who were receiving glucocorticoids alone or in combination with other immunosuppressants. Data on demographics, vital signs, laboratory results, and comorbidities were collected to assess the link between NLR and 90-day mortality. Multivariable Cox hazard regression analyses and smooth curve fitting were employed to assess the independent association between NLR and 90-day mortality. A two-piecewise linear regression model was used to examine the nonlinear association between NLR and in-hospital mortality. Receiver-operating characteristic curves (ROC) and area under the curves (AUC) were used to assess the ability of different biomarkers to predict the 90-day mortality in patients with pneumonia.

**Results:**

In total, 696 patients with pneumonia were included in this study. There were 332 individuals (47.7%) aged 18–59 years and 364 (52.3%) aged 60–99 years; 52.6% were male. The 90-day mortality rate across the study population was 26.1%. A non-linear association was noted between NLR and 90-day mortality, with an inflection point at approximately 16.475. On the left side of the inflection point, the hazard ratio was 1.145(95% confidence interval [CI]: 1.091–1.2, *p < *0.001). On the right side of the inflection point, the hazard ratio was 1.0057(95% CI:0.9923–1.0192; *p* = 0.406), reflecting a lack of statistical significance. Similar patterns were observed in subgroup analyses, with significant interaction effects noted for age and smoking status. Furthermore, the ROC curve analysis revealed that NLR was the optimal biomarker for predicting the 90-day mortality with an AUC of 0.714 (95% CI:0.670–0.757). Using 9.34 as the cutoff value of NLR, the sensitivity was 69.8%, and the specificity was 67.7%.

**Conclusions:**

A nonlinear correlation between NLR and 90-day mortality was identified in pneumonia patients undergoing glucocorticoid treatment. The NLR value of 16.475 represented the optimal threshold for predicting the 90-day mortality, after exceeding the threshold,90-day mortality tended to stabilize. The findings suggest that NLR is a practical and useful biomarker for predicting the 90-day mortality in this population.

## Introduction

Extended administration of high-dose glucocorticoids is associated with significant immunosuppression and increased susceptibility to severe infections, particularly pulmonary infections, which are a predominant cause of morbidity and mortality in this immunocompromised population [1]. The mortality rate from pulmonary infections in patients receiving prolonged glucocorticoid therapy approximates 45% [1–4], with strikingly similar mortality patterns observed in patients presenting with other etiologies of immunosuppression [5]. Globally, lower respiratory tract infections represent a substantial public health concern, with epidemiological data from 2019 documenting 489 million reported cases [6]. In the United States, pneumonia remains the ninth leading cause of mortality and the most prevalent infectious disease-related cause of death, resulting in an estimated 50,000 annual fatalities [7].

Given the high incidence and mortality of pulmonary infections in immunocompromised patients, identifying reliable biomarkers to predict the severity and outcomes of these infections is crucial. Because neutrophils and lymphocytes represent the largest percentage of all immune cells circulating in the bloodstream, the neutrophil-to-lymphocyte ratio (NLR), a hematological parameter that reflects systemic immune status and inflammatory responses, has recently emerged as a promising biomarker owing to its cost-effectiveness, rapid assessment, and widespread clinical availability, particularly in the context of chronic disease management [8–11]. NLR is calculated as the ratio of neutrophil count to lymphocyte count, typically from a peripheral blood sample. Under pathological stress conditions, neutrophil counts demonstrate a marked elevation, whereas lymphocyte populations show a substantial decline. Elevated NLR values have been observed in various infectious diseases, including acute exacerbations of chronic obstructive pulmonary disease (COPD) [12], pneumonia [13], coronavirus disease 2019 (COVID-19) [14,15], and sepsis [16], and have been associated with more severe disease and increased mortality risk [17,18].

Despite these findings, the association between NLR and mortality risk in patients with pneumonia receiving glucocorticoids remains unclear. Therefore, this study aimed to investigate the potential correlation between the NLR and 90-day mortality within this specific patient cohort and hypothesized that a higher NLR is associated with increased mortality in patients with pneumonia.

## Materials and Methods

### Study population

Dryad is an open data knowledge base that stores medical, biological, and ecological data. The data used in this study were derived from an observational study conducted in six secondary and tertiary academic hospitals in China [19], supplemented by a dataset obtained from the Dryad Digital Repository (https://doi.org/10.5061/dryad.mkkwh70×2). The Institutional Review Board of the China-Japan Friendship Hospital (Approval No.2015−86) authorized this retrospective investigation and oversaw centralized coordination among the participating institutions while ensuring standardized protocols for anonymized data collection and submission. Informed consent was waived due to the retrospective nature of the study.

The study enrolled 696 consecutive patients diagnosed with pneumonia who were hospitalized across six secondary and tertiary academic medical centers in China between January 1, 2013, and December 31, 2017. This cohort study adhered to the Strengthening the Reporting of Observational Studies in Epidemiology (STROBE) guidelines, ensuring rigorous methodological standards and transparent reporting of observational research [20].

The diagnosis of pneumonia was established in accordance with the joint guidelines issued by the American Thoracic Society and Infectious Diseases Society of America [21,22]. Diagnostic criteria encompassed the presence of newly identified pulmonary infiltrates on chest radiography or computed tomography (CT), combined with at least one of the following clinical features: (1) acute onset or exacerbation of respiratory symptoms, including productive cough with purulent sputum, with or without associated chest pain; (2) documented fever (axillary temperature ≥ 37.3°C) or hypothermia (axillary temperature<36°C); (3) physical examination findings suggestive of pulmonary consolidation and/or auscultatory crackles; or (4) leukocyte count abnormalities(>10 × 10^9^/L or <4 × 10^9^/L), irrespective of neutrophil predominance. We included patients presenting with connective tissue diseases, idiopathic interstitial pneumonia, nephrotic syndrome, chronic glomerulonephritis, COPD, bronchial asthma, or those undergoing alternative immunosuppressive therapies.

Patients were selected for the study based on the following inclusion criteria: (1) treatment with oral or intravenous corticosteroids treatment [4,23,24] prior to admission;(2) confirmed pneumonia diagnosis at admission or during the hospital stay; (3) aged ≥16 years at enrollment. The exclusion criteria were as follows: (1) presence of noninfectious pulmonary pathologies, including malignant pulmonary neoplasms, noninfectious interstitial pulmonary disorders, pulmonary thromboembolism, or congestive cardiac failure; and (2) unable to provide informed consent for procedures, such as bronchoalveolar lavage(BAL).

### Data collection

A multidisciplinary team of principal investigators, comprising clinical specialists, biostatisticians, microbiologists, and radiologists, collaboratively designed the study protocol and implemented a standardized case report form (CRF) to ensure methodological consistency across all participating centers. Before the study began, all investigators received training on the protocol, including the screening process, disease definitions, and CRF use. After the data collection, a trained researcher reviewed the CRFs to ensure completeness and quality.

A comprehensive set of clinical data was systematically collected from hospitalized patients’ medical records, encompassing:(1) demographic characteristics, including age and sex; (2) clinical manifestations, such as fever, cough, and dyspnea; (3) comorbidities, including asthma, COPD, coronary heart disease (CHD), diabetes mellitus (DM), and chronic renal failure; (4) disease severity, assessed using the Pneumonia Severity Index (PSI) score; (5) laboratory findings, including platelet count, aspartate aminotransferase (AST) levels, creatinine levels, and sodium (Na) levels; and (6) survival status at 30 and 90 days after admission.

### Statistical analysis

Patients were grouped into quartiles according to the NLR distribution. The baseline characteristics were summarized using descriptive statistical methods. For continuous variables exhibiting normal distribution, the data are presented as mean ± standard deviation (SD), whereas those with skewed distributions are described using median and interquartile range (IQR). To evaluate the differences among the groups, categorical variables were analyzed using either the chi-squared test or Fisher’s exact test. Normally distributed continuous variables were examined using one-way analysis of variance (ANOVA), whereas non-normally distributed continuous variables were evaluated using the Kruskal-Wallis H test.

Cox regression analyses were performed to examine the relationship between NLR and mortality within 90 days. Multiple adjusted models were developed, with the extended Cox model applied, utilizing the lowest quartile of NLR as the reference category. Model 1 represented the crude analysis without any adjustments. Model 2 incorporated adjustments for demographic variables including age and sex. Model 3 included the key clinical comorbidities of asthma, COPD, CHD, DM, and CRF. The final and most comprehensive model (Model 4) was adjusted for all aforementioned variables, in addition to lifestyle factors (smoking status and alcohol consumption), laboratory parameters(platelet count [PLT], aspartate aminotransferase [AST], creatinine [CRE], and serum sodium [Na]), and disease severity as measured by the PSI.

In addition, to investigate the dose-response association linking NLR to mortality within 90 days, smooth curve fitting was implemented. A likelihood ratio test was conducted to compare the two-piecewise linear model with the one-line linear regression model. Survival outcomes were analyzed using Kaplan–Meier survival curves, with stratification by NLR quartiles and statistical significance assessed via the log-rank test. To evaluate the robustness of the NLR-mortality link, stratified and interaction analyses were carried out across multiple subgroups, encompassing demographic factors (age, sex), comorbid conditions (asthma, COPD, CHD, DM), and behavioral factors (smoking status, alcohol consumption). Additionally, a sensitivity analysis was performed using patients with an NLR between 0.022 and 3.65 as the reference group. Patients with an NLR above 3.65 were further categorized into three groups: mild NLR (3.65–7.66), moderate NLR (7.66–15.46), and severe NLR (15.46–222.92). The Youden index was applied to determine the optimal cut-off value for each variable, and receiver operating characteristic (ROC) curve analysis assessed the sensitivity and specificity of different biomarkers predicting adverse prognosis. The area under the curve (AUC) was used to evaluate prognostic accuracy, with comparisons conducted via a nonparametric approach. Higher AUC values indicate greater discriminatory ability. To assess the robustness of our findings, sensitivity analysis was conducted by reclassifying NLR according to the established optimal cutoff value of 10.0, derived from previous studies [17,32]. We used multiple imputations, based on 5 replications and a chained equation approach method in the R MI procedure, to maximize statistical power and minimize bias that might occur to account for missing data [25]. The details of the missing value are shown in S1 Table.

All statistical analyses were conducted using R Statistical Software (version 4.2.2, http://www.R-project.org, The R Foundation) and Free Statistics Analysis Platform (version 2.1.1, Beijing, China, http://www.clinicalscientists.cn/freestatistics) [26]. A two-tailed test was used to determine statistical significance, with the threshold set at *p* < 0.05.

## Results

### Participants’ characteristics

**Fig 1:**
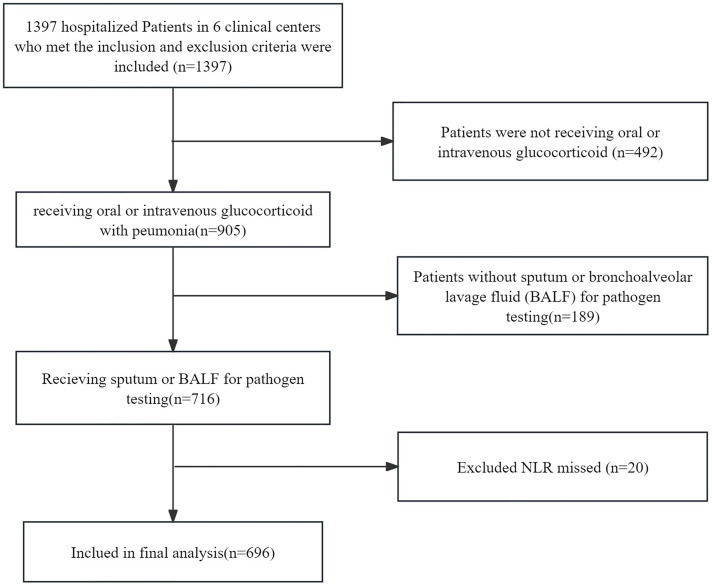
Flow chart of the study.

Initially, there were 716 patients with pneumonia receiving glucocorticoids in the Dryad database. After excluding missing values, 696 patients were included in the study, with 332 (47.7%) aged between 18 and 59 years, and 364 (52.3%) aged between 60 and 99 years (see Fig 1 for details). Additionally, 52.6% of the participants were male. The predominant clinical manifestations observed in the study cohort were cough (87.8%), followed by fever (74.6%) and dyspnea (60.3%), respectively. The 90-day mortality rate for the patients was 26.1%. The baseline characteristics of all participants are shown in Table 1.

**Table 1:** Baseline and clinical characteristics of the study population.

Variables	Total (n = 696)	Q1(0.02–3.64) (n = 174)	Q2(3.65–7.65) (n = 174)	Q3(7.66–15.45) (n = 174)	Q4(15.46–222.92) (n = 174)	*P* value
NLR, Median (IQR)	0.8 (0.4, 1.5)	0.2 (0.2, 0.3)	0.6 (0.5, 0.7)	1.1 (0.9, 1.3)	2.4 (1.8, 3.3)	<0.001
Age, n (%)						0.066
18–59	332 (47.7)	73 (42)	83 (47.7)	97 (55.7)	79 (45.4)	
60–99	364 (52.3)	101 (58)	91 (52.3)	77 (44.3)	95 (54.6)	
Sex, n (%)						0.342
Male	366 (52.6)	85 (48.9)	86 (49.4)	97 (55.7)	98 (56.3)	
Female	330 (47.4)	89 (51.1)	88 (50.6)	77 (44.3)	76 (43.7)	
Asthma, n (%)						0.133
No	679 (97.6)	168 (96.6)	167 (96)	171 (98.3)	173 (99.4)	
Yes	17 (2.4)	6 (3.4)	7 (4)	3 (1.7)	1 (0.6)	
COPD, n (%)						0.206
No	595 (85.5)	141 (81)	148 (85.1)	152 (87.4)	154 (88.5)	
Yes	101 (14.5)	33 (19)	26 (14.9)	22 (12.6)	20 (11.5)	
CHD, n (%)						0.537
No	610 (87.6)	150 (86.2)	149 (85.6)	157 (90.2)	154 (88.5)	
Yes	86 (12.4)	24 (13.8)	25 (14.4)	17 (9.8)	20 (11.5)	
DM, n (%)						0.358
No	523 (75.1)	125 (71.8)	130 (74.7)	139 (79.9)	129 (74.1)	
Yes	173 (24.9)	49 (28.2)	44 (25.3)	35 (20.1)	45 (25.9)	
CRF, n (%)						0.473
No	643 (92.4)	163 (93.7)	162 (93.1)	156 (89.7)	162 (93.1)	
Yes	53 (7.6)	11 (6.3)	12 (6.9)	18 (10.3)	12 (6.9)	
Smoke, n (%)						0.691
Never smoke	509 (73.1)	130 (74.7)	120 (69)	133 (76.4)	126 (72.4)	
Former smoke	160 (23.0)	36 (20.7)	46 (26.4)	35 (20.1)	43 (24.7)	
Current smoke	27 (3.9)	8 (4.6)	8 (4.6)	6 (3.4)	5 (2.9)	
Alcoholism, n (%)						0.021
No	639 (91.8)	163 (93.7)	159 (91.4)	166 (95.4)	151 (86.8)	
Yes	57 (8.2)	11 (6.3)	15 (8.6)	8 (4.6)	23 (13.2)	
PLT × 10^9^/L	190.5 ± 90.7	179.7 ± 86.0	202.6 ± 90.0	196.1 ± 93.5	183.5 ± 92.1	0.064
AST(U/L)	24.0 (16.0, 39.0)	20.6 (16.0, 28.8)	22.0 (16.0, 37.0)	25.0 (16.7, 43.5)	27.0 (18.0, 43.8)	0.001
CRE(μmmol/L)	64.0 (50.9, 90.3)	63.4 (53.3, 82.6)	63.4 (49.0, 87.6)	63.2 (49.8, 92.0)	68.1 (49.6, 106.6)	0.537
Na(mmol/L)	137.6 ± 7.5	139.5 ± 8.5	138.1 ± 9.2	137.0 ± 5.2	135.8 ± 5.9	< 0.001
PSI	81.1 ± 31.6	73.7 ± 27.3	78.1 ± 30.6	79.2 ± 31.1	93.3 ± 33.7	< 0.001

Abbreviations: COPD, chronic obstructive pulmonary disease; CHD, coronary heart disease; DM, diabetes mellitus; CRF, coronary renal failure; PLT, platelet; AST, Aspartate Aminotransferase; CRE, creatinine; PSI, pneumonia severity index (PSI) score.

### Association between NLR and mortality

To examine the association between NLR and 90-day mortality in patients with pneumonia, we constructed a series of multivariate regression models with progressive adjustments (Table 2). Model 1 was unadjusted, whereas Model 2 was adjusted for age and sex. Model 3 was additionally adjusted for comorbidities. The fully adjusted Model 4 included all the aforementioned variables, along with smoking status, alcohol consumption, PLT, AST, CRE, Na levels, and PSI (Table 2).

**Table 2:** Relationship between neutrophil-to-lymphocyte ratio and 90-mortality in different models.

Variable	Model 1		Model 2		Model 3		Model 4	
	HR (95%CI)	*P* value	HR (95%CI)	*P* value	HR (95%CI)	*P* value	HR (95%CI)	*P* value
NLR count per 10	1.14 (1.1–1.18)	<0.001	1.14 (1.1 ~ 1.19)	<0.001	1.13 (1.09 ~ 1.18)	<0.001	1.11 (1.06 ~ 1.16)	<0.001
Q1(0.02–3.64)	1(Ref)		1(Ref)		1(Ref)		1(Ref)	
Q2(3.65–7.65)	2.14 (1.15–3.96)	0.016	2.16 (1.17 ~ 4.01)	0.014	2.15 (1.16 ~ 3.99)	0.015	2.01 (1.08 ~ 3.75)	0.029
Q3(7.66–15.45)	3.77 (2.12–6.7)	<0.001	3.93 (2.2 ~ 7)	<0.001	3.94 (2.21 ~ 7.03)	<0.001	3.74 (2.08 ~ 6.73)	<0.001
Q4(15.46–222.92)	7.58 (4.37–13.12)	<0.001	7.6 (4.39 ~ 13.18)	<0.001	7.38 (4.25 ~ 12.8)	<0.001	6.06 (3.44 ~ 10.7)	<0.001
*P* for Trend	1.93 (1.67–2.24)	<0.001	1.93 (1.66 ~ 2.24)	<0.001	1.91 (1.64 ~ 2.21)	<0.001	1.78 (1.53 ~ 2.08)	<0.001

NLR, Neutrophil-to-Lymphocyte Ratio.

Model 1: Unadjusted.

Model 2: Adjusted for age, sex.

Model 3: Adjusted for age, sex, asthma, COPD, CHD, DM and CRF.

Model 4: Adjust for age, sex, asthma, COPD, CHD, DM, CRF, smoke, alcoholism, PLT, AST, CRE, Na and PSI.

As a continuous variable, in the fully adjusted multivariate regression model (Model 4), the 90-day mortality increased by 11% for every 10-unit increase in NLR(hazard ratio[HR]=1.11,95% confidence interval [CI]=1.06–1.16, *p* < 0.001).

When categorized into NLR quartile, comprehensive multivariate regression analysis (Model 4) revealed a persistent significant association with 90-day mortality. Patients in the highest quartile (Q4) exhibited a substantially elevated 90-day mortality compared with those in the lowest quartile (Q1) (Q4: HR = 6.06,95% CI:3.44–10.7, *p* < 0.001). Notably, all four models demonstrated statistically significant trend associations (*p* < 0.05).

### Nonlinearity relationship between NLR and mortality

**Fig 2:**
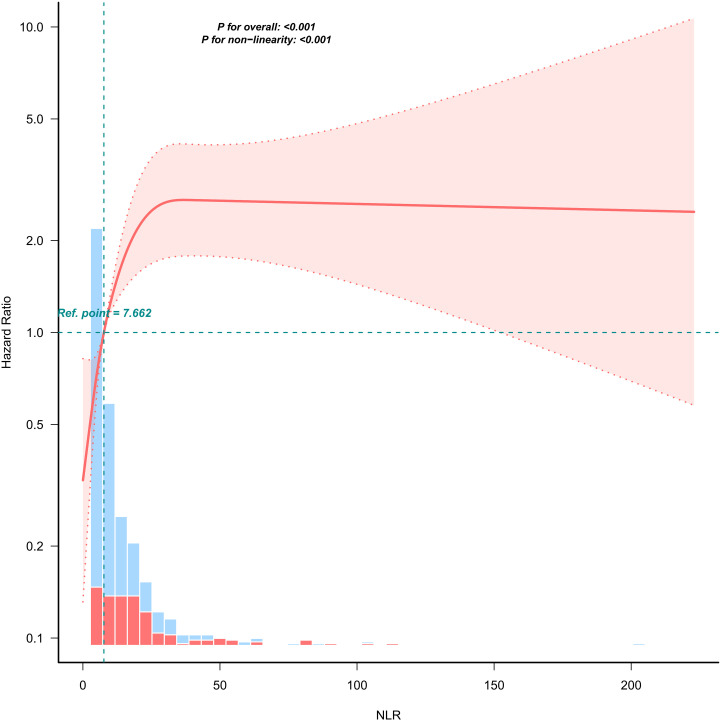
Dose-response relationship between Neutrophil-to-Lymphocyte ratio and 90-day mortality. Adjusted for age, sex, asthma, COPD, CHD, DM, CRF, smoke, alcoholism, PLT, AST, CRE, Na, and PSI.

We revealed a non-linear relationship between the NLR and 90-day mortality, as determined through the application of a multivariate Cox hazard regression model and smooth curve fitting (Fig 2). Given the significant nonlinearity (*p* < 0.001) (Table 3), we utilized a two-piecewise linear model to characterize the relationship between NLR and 90-day mortality; a significant inflection point was identified at 16.475 (see Fig 2). To the left of this threshold, the HR was 1.145 (95% CI 1.091–1.2, *p* < 0.001), indicating a positive association; conversely, to the right of the inflection point, the HR exhibited a non-significant decline to 1.0057 (95% CI 0.9923–1.0192, *p* = 0.4064). Notably, the association on the right side of the inflection point was not statistically significant, suggesting that for NLR values below approximately 16.475, every 1 unit increase in NLR conferred a 14.5% higher in 90-day mortality. Furthermore, beyond this threshold,90-day mortality demonstrated stabilization, but still high.

**Table 3:** The non-linear relationship between Neutrophil-to-Lymphocyte ratio and 90-day mortality.

Threshold of Neutrophil-to-Lymphocyte ratio	HR	95%CI	*P* value
<16.475	1.145	1.145 (1.091,1.2)	< 0.001
≥16.475	1.0057	1.0057 (0.9923,1.0192)	0.4064
Likelihood Ratio test			<0.001

Abbreviations: HR, hazard ratio; CI, confidence interval. HRs were adjusted for age, sex, asthma, COPD, CHD, DM, CRF, smoke, alcoholism, PLT, AST, CRE, Na and PSI. Only 99.5% of the data is displayed.

### Kaplan–Meier survival curve analysis

**Fig 3:**
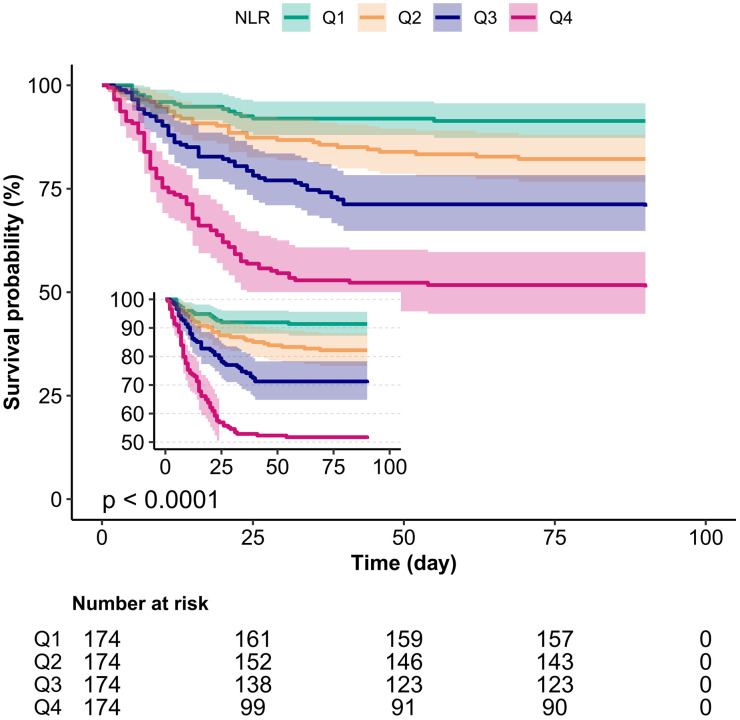
Kaplan–Meier survival curves for day 90 of patients with pneumonia depending on the quartile of neutrophil-to-lymphocyte ratio. Adjusted for age, sex, asthma, COPD, CHD, DM, CRF, smoke, alcoholism, PLT, AST, CRE, Na and PSI.

As depicted in the Kaplan–Meier curve (Fig 3), the 90-day cumulative survival rate of the Q4 group was significantly lower than that of the other groups (*p < *0.0001).

### Subgroup analyses

**Fig 4:**
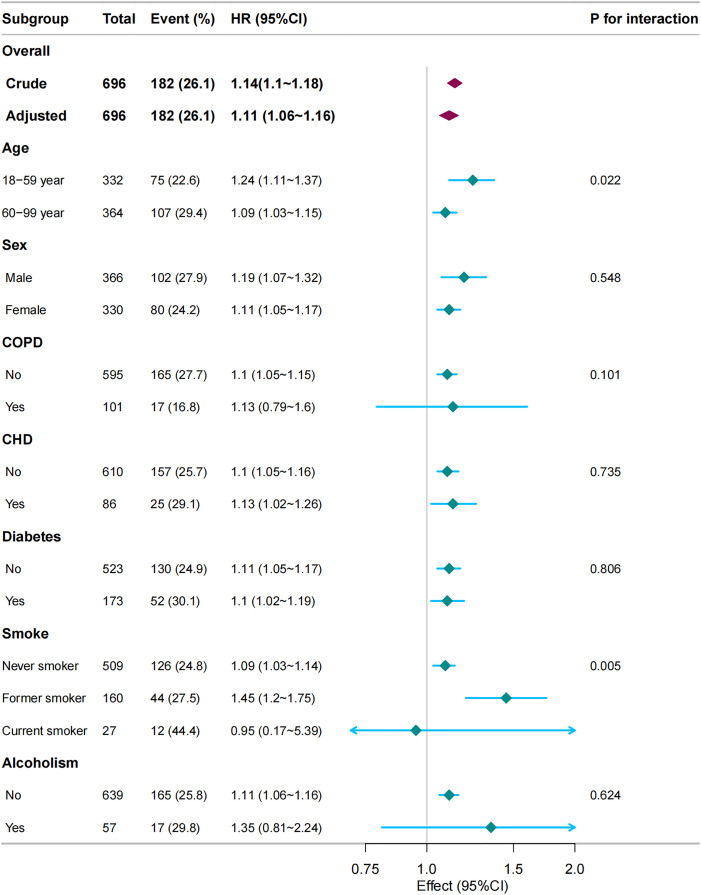
Subgroup and stratified analyses of the association between Neutrophil-to-Lymphocyte ratio with 90-day mortality. Adjusted for age, sex, asthma, COPD, CHD, DM, CRF, smoke, alcoholism, PLT, AST, CRE, Na and PSI.

Stratified and interaction analyses were conducted to evaluate the consistency of the association between the NLR and 90-day mortality across diverse subgroups, including age, sex, asthma, COPD, CHD, DM, smoking status, and alcohol consumption. The forest plot revealed a robust and independent association between the NLR and 90-day mortality in patients with pneumonia (Fig 4). Notably, significant interaction effects were identified for age and smoking status in relation to the NLR-mortality association (*p* < 0.05).

### Diagnostic efficiency of NLR compared with other biomarkers

We further assessed the prognostic value of NLR in patients with pneumonia. ROC curves generated for NLR and established biomarkers were illustrated in S1 Fig. The AUC value for NLR was 0.714. For predicting 90-day mortality, NLR demonstrated superior performance relative to established biomarkers, including WBC, NEUT, LYM, CRP, PCT(AUC_WBC_ = 0.607, AUC_NEUT_ = 0.653, AUC_LYM_ = 0.662, AUC_CRP_ = 0.529, AUC_PCT_ = 0.564, *p* < 0.05). Using 9.34 as the optimal cutoff value of NLR, the sensitivity was 69.78%, and the specificity was 67.70% (S2 Table).

### Sensitivity analysis

For sensitivity analysis, the NLR was dichotomised using the optimal cutoff value of 10.0, previously established via ROC curve analysis, confirming a persistent association between NLR and 90-day mortality in patients with pneumonia (HR: 3.15, 95% CI 2.29–4.32, *p* < 0.001), as detailed in S3 Table.

## Discussion

This study demonstrates a significant association between the NLR and 90-day mortality in patients with pneumonia treated with glucocorticoids. The findings demonstrate a statistically significant elevation in 90-day mortality associated with higher NLR values. Interestingly, the threshold effect curve analysis revealed a clear association between NLR and 90-day mortality below the inflection point. An increase of 1 unit in NLR is linked to a 14.5% increase in mortality when NLR is 16.475. Notably, there was evidence suggesting a dose-response relationship, indicating that the association between NLR and 90-day mortality exhibited a nonlinear pattern (*p* for nonlinearity <0.001). The NLR, derived from routine complete blood counts, serves as an inflammation biomarker reflecting systemic immune dysregulation. Elevated NLR values exhibit significant association with 90-day mortality in patients with pneumonia, necessitating heightened clinical attention to inflammatory status. Its utility as a rapid, cost-effective, and widely accessible biomarker is particularly valuable in severe pneumonia, enabling prompt assessment of inflammatory burden and may provide clinicians with quick stratification. This finding suggests that elevated NLR may portend adverse hospital outcomes, potentially indicative of greater disease severity and suboptimal anti-inflammatory treatment response. Specifically, an NLR threshold ≥16.475 demonstrates significant predictive value for 90-day mortality in this population. Both the sensitivity and stratified analyses confirmed that the association between NLR and 90-day mortality remained robust. In stratified analyses, significant interaction effects were observed for age and smoking status but not for other variables. The potential interaction effects of age and smoking on the association between NLR and 90-day mortality in patients with pneumonia warrant further investigation.

The host inflammatory response is a pivotal factor in the development and progression of pneumonia. Noninvasive inflammatory biomarkers, including C-reactive protein (CRP), erythrocyte sedimentation rate (ESR), white blood cell (WBC) count, procalcitonin, interleukin (IL)-6, IL-8, interferon-alpha, and tumor necrosis factor-alpha, have been extensively utilized to enhance diagnostic accuracy. This is because the outcomes of specimen cultures as well as laboratory and radiologic assessments are not invariably informative. However, the majority of these biomarkers are costly and, as a result, are not consistently employed in routine clinical practice. Consequently, there remains an unmet need for novel biomarkers that are simple, specific, and cost-effective for the diagnosis and monitoring of pneumonia. Inflammatory biomarkers, including NLR, systemic immune-inflammation index (SII), systemic inflammation response index (SIRI), and platelet-to-lymphocyte ratio (PLR), can be used to evaluate the status of systemic inflammation and characteristics of immune response [27]. Neutrophilia and lymphocytopenia represent characteristic physiological manifestations of the innate immune system in response to systemic inflammatory processes. The pathophysiology of lymphocytopenia involves three distinct mechanisms: (1) enhanced programmed cell death of lymphocytes;(2) sequestration of these cells within the reticuloendothelial system, hepatic tissues, and visceral lymphatic networks; and (3) altered trafficking patterns throughout the lymphatic system. Neutrophilia represents the converse phenomenon during systemic inflammation, resulting from the demargination of neutrophils and the stimulation of stem cells by growth factors, specifically granulocyte-colony stimulating factor [28–30]. The NLR, which is taken in routine blood tests and can reveal the imbalance between pro-inflammatory and anti-inflammatory systems, has broad application possibilities in assessing inflammatory reactions and prognosis. It has been reported that elevated NLR serves as a reliable biomarker reflecting both disease severity and prognostic outcomes in patients with severe infections or systemic inflammatory conditions [31].

NLR has emerged as a pivotal biomarker for evaluating systemic inflammatory responses and infection susceptibility, demonstrating significant prognostic value in various clinical contexts including community-acquired pneumonia [17,32], infectious diseases [33], and intracerebral hemorrhage-related stroke outcomes [34]. Recent clinical investigations have demonstrated that the NLR exhibits superior prognostic accuracy compared with conventional infection biomarkers, including CRP, WBC count, and neutrophil count, in young adult patients presenting with community-acquired pneumonia in emergency department settings [17]. Gunay et al. pioneered the clinical application of the NLR as an inflammatory severity biomarker in patients with COPD [35], while subsequent studies further identified NLR as an independent prognostic indicator for both COPD exacerbation severity and mortality risk [36,37]. Notably, a comprehensive systematic review and meta-analysis demonstrated that NLR is significantly associated with multiple clinical outcomes in patients with acute exacerbation of COPD, encompassing clinical symptom profiles and pulmonary function parameters as well as prognostic indicators such as increased risk of bacterial infection and both short-(90-day) and long-term (24-month) mortality [38]. Xiaoyi Feng [12] demonstrated that an NLR > 14.17 at discharge was an independent risk factor for 90-day mortality in hospitalized patients with acute exacerbation of COPD, which was consistent with our study’s findings, emphasizing that the NLR serves as a crucial indicator for predicting outcomes individuals diagnosed with pneumonia. The results obtained in our investigation demonstrate consistency with those reported in previous studies. Since the association between NLR and mortality in pneumonia patients has been extensively studied [17,18,32], we compared the predictive value of NLR with currently biomarkers in predicting 90-day mortality in pneumonia patients. We found that NLR performed better than WBC, NEUT, LYM, CRP and PCT.

In addition to these widely acknowledged findings, several noteworthy observations have emerged from our study. First, we consistently identified an inverted L-shaped association between NLR and 90-day mortality in patients with pneumonia taking glucocorticoids, even after adjusting for potential confounders. Consequently, elevated NLR may serve as a valuable predictor of the 90-day mortality in this population. Importantly, the magnitudes of the significant associations between NLR and incident 90-day mortality in patients with pneumonia taking glucocorticoids were also consistent across subgroups stratified by age, sex, asthma, COPD, CHD, DM, smoking, and alcohol consumption; age and smoking status had significant interaction effects(*p* < 0.05).

Our study has several strengths and presents two significant methodological contributions to the field of pneumonia research. Primarily, it represents a pioneering investigation into the prognostic significance of the NLR in patients with pneumonia. We also employed advanced smooth curve fitting techniques to elucidate potential nonlinear associations between NLR levels and clinical outcomes, thereby providing a more nuanced understanding of this relationship. Furthermore, to meticulously control for potential confounding variables, we employed a robust Cox regression analysis. This approach entailed the integration of multiple models to comprehensively assess the data. In addition, we conducted subgroup analyses using meticulously defined categories to further elucidate the relationships under investigation.

However, this study has several limitations that must be acknowledged. First, due to the nature of the observational study, we only examined the correlation of NLR with 90-day mortality, and could not establish the cause-effect relationship between them. Second, despite performing regression, stratified analysis, there is still a possibility of residual confounding from unmeasured or unknown factors. Third, the limited sample size in our study may have restricted the comprehensive evaluation of statistical power and the exploration of potential interrelationships among multiple variables. While these preliminary observations may provide clinically relevant insights, external validation through larger, well-designed cohort studies is essential to establish robust evidence and strengthen the reliability of our findings. Moreover, while our findings are primarily confined to hospitalized pneumonia patients undergoing glucocorticoid-based regimens, the identified inflection point may possess broader applicability to the general population of hospitalized pneumonia patients and external validation of the 16.475 threshold will be performed in subsequent studies. Finally, it is important to note that our study predominantly included participants from China, which limits the generalizability of our results and underscores the need for parallel studies in diverse populations. In the future, prospective cohort studies or subgroups based on randomized controlled trials will be conducted to strengthen causal inference, including the use of external validate cohort to confirm our findings.

## Conclusions

We have shown that the NLR is a predictor of 90-day mortality in patients with pneumonia. A non-linear relationship was identified, indicating that high levels of NLR are associated with an increased 90-day mortality in this population, with a threshold value of 16.475. The findings presented herein warrant attention, as this association may be crucial for clinicians when considering treatment strategies aimed at reducing 90-day mortality in patients with pneumonia. It may provide clinicians with quick stratification of patients into different prognostic categories. Given the potential for confounding factors, further studies are warranted to corroborate these results. This study should serve as the foundation for future well-designed studies to evaluate the effect of elevated NLR on mortality and establish potential causal relationships.

## Supporting information

S1 Fig ROC curves. (TIF)

S1 Table Details of missing values. Abbreviations: COPD, chronic obstructive pulmonary disease; CHD, coronary heart disease; DM, diabetes mellitus; CRF, coronary renal failure; PLT, platelet; AST, Aspartate Aminotransferase; CRE, creatinine; PSI, pneumonia severity index (PSI) score.
(TIF)

S2 Table Cut-Off points of NLR, CRP, PCT, WBC, LYM and NEUT for 90-day mortality. NLR: Neutrophil/Lymphocyte Ratio; CRP: C-Reactive Protein; PCT: Procalcitonin; WBC: White Blood Cell Count; LYM: Lymphocyte Count; NEUT: Neutrophil Count; NPV: negative predictive value; PPV: positive predictive value; AUC: area-under-curve.
(DOCX)

S3 Table Sensitivity analysis. NLR, Neutrophil-to-Lymphocyte Ratio. Model 1: unadjusted. Model 2: adjusted for age, sex. Model 3: adjusted for age, sex, asthma, COPD, CHD, DM and CRF. Model 4 adjust for age, sex, asthma, COPD, CHD, DM, CRF, smoke, alcoholism, PLT, AST, CRE, Na and PSI.
(DOCX)

## References

[1] Agustí C, Rañó A, Filella X, González J, Moreno A, Xaubet A, et al. Pulmonary infiltrates in patients receiving long-term glucocorticoid treatment: etiology, prognostic factors, and associated inflammatory response. Chest. 2003;123(2):488–98. doi: 10.1378/chest.123.2.488 12576371

[2] Ajmal S, Mahmood M, Abu Saleh O, Larson J, Sohail MR. Invasive fungal infections associated with prior respiratory viral infections in immunocompromised hosts. Infection. 2018;46(4):555–8. doi: 10.1007/s15010-018-1138-0 29627936

[3] Fillâtre P, Revest M, Belaz S, Robert-Gangneux F, Zahar J-R, Roblot F, et al. Pneumocystosis in non-HIV-infected immunocompromised patients. Rev Med Interne. 2016;37(5):327–36. doi: 10.1016/j.revmed.2015.10.002 26644039

[4] Kofteridis DP, Valachis A, Velegraki M, Antoniou M, Christofaki M, Vrentzos GE, et al. Predisposing factors, clinical characteristics and outcome of Pneumonocystis jirovecii pneumonia in HIV-negative patients. J Infect Chemother. 2014;20(7):412–6. doi: 10.1016/j.jiac.2014.03.003 24767467

[5] Rañó A, Agustí C, Jimenez P, Angrill J, Benito N, Danés C, et al. Pulmonary infiltrates in non-HIV immunocompromised patients: a diagnostic approach using non-invasive and bronchoscopic procedures. Thorax. 2001;56(5):379–87. doi: 10.1136/thorax.56.5.379 11312407 PMC1746047

[6] GBD 2019 LRI Collaborators. Age-sex differences in the global burden of lower respiratory infections and risk factors, 1990-2019: results from the Global Burden of Disease Study 2019. Lancet Infect Dis. 2022;22(11):1626–47. doi: 10.1016/S1473-3099(22)00510-2 35964613 PMC9605880

[7] Heron M. Deaths: Leading Causes for 2019. Natl Vital Stat Rep. 2021;70(9):1–114. 34520342

[8] El-Gazzar AG, Kamel MH, Elbahnasy OKM, El-Naggar ME-S. Prognostic value of platelet and neutrophil to lymphocyte ratio in COPD patients. Expert Rev Respir Med. 2020;14(1):111–6. doi: 10.1080/17476348.2019.1675517 31577911

[9] Luo Z, Zhang W, Chen L, Xu N. Prognostic Value of Neutrophil: Lymphocyte and Platelet: Lymphocyte Ratios for 28-Day Mortality of Patients with AECOPD. Int J Gen Med. 2021;14:2839–48. doi: 10.2147/IJGM.S312045 34211292 PMC8242126

[10] Paliogiannis P, Fois AG, Sotgia S, Mangoni AA, Zinellu E, Pirina P, et al. The neutrophil-to-lymphocyte ratio as a marker of chronic obstructive pulmonary disease and its exacerbations: A systematic review and meta-analysis. Eur J Clin Invest. 2018;48(8):e12984. doi: 10.1111/eci.12984 29924383

[11] Sørensen AK, Holmgaard DB, Mygind LH, Johansen J, Pedersen C. Neutrophil-to-lymphocyte ratio, calprotectin and YKL-40 in patients with chronic obstructive pulmonary disease: correlations and 5-year mortality - a cohort study. J Inflamm (Lond). 2015;12:20. doi: 10.1186/s12950-015-0064-5 25908927 PMC4407303

[12] Feng X, Xiao H, Duan Y, Li Q, Ou X. Prognostic Value of Neutrophil to Lymphocyte Ratio for Predicting 90-Day Poor Outcomes in Hospitalized Patients with Acute Exacerbation of Chronic Obstructive Pulmonary Disease. Int J Chron Obstruct Pulmon Dis. 2023;18:1219–30. doi: 10.2147/COPD.S399671 37337582 PMC10276987

[13] Kakehi E, Uehira R, Ohara N, Akamatsu Y, Osaka T, Sakurai S, et al. Utility of the New Early Warning Score (NEWS) in combination with the neutrophil-lymphocyte ratio for the prediction of prognosis in older patients with pneumonia. Fam Med Community Health. 2023;11(2):e002239. doi: 10.1136/fmch-2023-002239 37344123 PMC10314686

[14] Regolo M, Sorce A, Vaccaro M, Colaci M, Stancanelli B, Natoli G, et al. Assessing Humoral Immuno-Inflammatory Pathways Associated with Respiratory Failure in COVID-19 Patients. J Clin Med. 2023;12(12):4057. doi: 10.3390/jcm12124057 37373750 PMC10299383

[15] Liu J, Liu Y, Xiang P, Pu L, Xiong H, Li C, et al. Neutrophil-to-lymphocyte ratio predicts critical illness patients with 2019 coronavirus disease in the early stage. J Transl Med. 2020;18(1):206. doi: 10.1186/s12967-020-02374-0 32434518 PMC7237880

[16] Ru S, Luo Y. The association and prognostic value of systemic inflammatory response index with short and long-term mortality in patients with sepsis. Medicine (Baltimore). 2023;102(29):e33967. doi: 10.1097/MD.0000000000033967 37478261 PMC10662841

[17] de Jager CPC, Wever PC, Gemen EFA, Kusters R, van Gageldonk-Lafeber AB, van der Poll T, et al. The neutrophil-lymphocyte count ratio in patients with community-acquired pneumonia. PLoS One. 2012;7(10):e46561. doi: 10.1371/journal.pone.0046561 23049706 PMC3462173

[18] Cataudella E, Giraffa CM, Di Marca S, Pulvirenti A, Alaimo S, Pisano M, et al. Neutrophil-To-Lymphocyte Ratio: An Emerging Marker Predicting Prognosis in Elderly Adults with Community-Acquired Pneumonia. J Am Geriatr Soc. 2017;65(8):1796–801. doi: 10.1111/jgs.14894 28407209

[19] Li L, Hsu SH, Gu X, Jiang S, Shang L, Sun G, et al. Aetiology and prognostic risk factors of mortality in patients with pneumonia receiving glucocorticoids alone or glucocorticoids and other immunosuppressants: a retrospective cohort study. BMJ Open. 2020;10(10):e037419. doi: 10.1136/bmjopen-2020-037419 33109645 PMC7592294

[20] Seymour CW, Liu VX, Iwashyna TJ, Brunkhorst FM, Rea TD, Scherag A, et al. Assessment of Clinical Criteria for Sepsis: For the Third International Consensus Definitions for Sepsis and Septic Shock (Sepsis-3). JAMA. 2016;315(8):762–74. doi: 10.1001/jama.2016.0288 26903335 PMC5433435

[21] American Thoracic Society, Infectious Diseases Society of America. Guidelines for the management of adults with hospital-acquired, ventilator-associated, and healthcare-associated pneumonia. Am J Respir Crit Care Med. 2005;171(4):388–416. doi: 10.1164/rccm.200405-644ST 15699079

[22] Sousa D, Justo I, Domínguez A, Manzur A, Izquierdo C, Ruiz L, et al. Community-acquired pneumonia in immunocompromised older patients: incidence, causative organisms and outcome. Clin Microbiol Infect. 2013;19(2):187–92. doi: 10.1111/j.1469-0691.2012.03765.x 22390624

[23] Jouneau S, Poineuf J-S, Minjolle S, Tattevin P, Uhel F, Kerjouan M, et al. Which patients should be tested for viruses on bronchoalveolar lavage fluid?. Eur J Clin Microbiol Infect Dis. 2013;32(5):671–7. doi: 10.1007/s10096-012-1791-7 23238685 PMC7101843

[24] Silva DR, Menegotto DM, Schulz LF, Gazzana MB, Dalcin P de TR. Clinical characteristics and evolution of non-HIV-infected immunocompromised patients with an in-hospital diagnosis of tuberculosis. J Bras Pneumol. 2010;36(4):475–84. doi: 10.1590/s1806-37132010000400013 20835595

[25] van Buuren S, Groothuis-Oudshoorn K. mice: Multivariate Imputation by Chained Equations inR. J Stat Soft. 2011;45(3). doi: 10.18637/jss.v045.i03

[26] Yang Q, Chen W, Wen Y, Zheng J, Chen J, Yu S, et al. Association Between Wait Time of Central Venous Pressure Measurement and Outcomes in Critical Patients With Acute Kidney Injury: A Retrospective Cohort Study. Front Public Health. 2022;10:893683. doi: 10.3389/fpubh.2022.893683 36016902 PMC9395608

[27] Yan D, Dai C, Xu R, Huang Q, Ren W. Predictive Ability of Systemic Inflammation Response Index for the Risk of Pneumonia in Patients with Acute Ischemic Stroke. Gerontology. 2023;69(2):181–8. doi: 10.1159/000524759 35584610

[28] Ayala A, Herdon CD, Lehman DL, Ayala CA, Chaudry IH. Differential induction of apoptosis in lymphoid tissues during sepsis: variation in onset, frequency, and the nature of the mediators. Blood. 1996;87(10):4261–75. 8639785

[29] Hotchkiss RS, Swanson PE, Freeman BD, Tinsley KW, Cobb JP, Matuschak GM, et al. Apoptotic cell death in patients with sepsis, shock, and multiple organ dysfunction. Crit Care Med. 1999;27(7):1230–51. doi: 10.1097/00003246-199907000-00002 10446814

[30] Unsinger J, Kazama H, McDonough JS, Hotchkiss RS, Ferguson TA. Differential lymphopenia-induced homeostatic proliferation for CD4+ and CD8+ T cells following septic injury. J Leukoc Biol. 2009;85(3):382–90. doi: 10.1189/jlb.0808491 19088177 PMC2653946

[31] Zahorec R. Ratio of neutrophil to lymphocyte counts--rapid and simple parameter of systemic inflammation and stress in critically ill. Bratisl Lek Listy. 2001;102(1):5–14. 11723675

[32] Curbelo J, Luquero Bueno S, Galván-Román JM, Ortega-Gómez M, Rajas O, Fernández-Jiménez G, et al. Inflammation biomarkers in blood as mortality predictors in community-acquired pneumonia admitted patients: Importance of comparison with neutrophil count percentage or neutrophil-lymphocyte ratio. PLoS One. 2017;12(3):e0173947. doi: 10.1371/journal.pone.0173947 28301543 PMC5354424

[33] de Jager CPC, van Wijk PTL, Mathoera RB, de Jongh-Leuvenink J, van der Poll T, Wever PC. Lymphocytopenia and neutrophil-lymphocyte count ratio predict bacteremia better than conventional infection markers in an emergency care unit. Crit Care. 2010;14(5):R192. doi: 10.1186/cc9309 21034463 PMC3219299

[34] Zhang F, Ren Y, Fu W, Yang Z, Wen D, Hu X, et al. Predictive Accuracy of Neutrophil-to-Lymphocyte Ratio on Long-Term Outcome in Patients with Spontaneous Intracerebral Hemorrhage. World Neurosurg. 2019;125:e651–7. doi: 10.1016/j.wneu.2019.01.143 30716500

[35] Günay E, Sarınç Ulaşlı S, Akar O, Ahsen A, Günay S, Koyuncu T, et al. Neutrophil-to-lymphocyte ratio in chronic obstructive pulmonary disease: a retrospective study. Inflammation. 2014;37(2):374–80. doi: 10.1007/s10753-013-9749-1 24078279

[36] Teng F, Ye H, Xue T. Predictive value of neutrophil to lymphocyte ratio in patients with acute exacerbation of chronic obstructive pulmonary disease. PLoS One. 2018;13(9):e0204377. doi: 10.1371/journal.pone.0204377 30265703 PMC6161875

[37] Wang H, Lv C, Wang S, Ying H, Weng Y, Yu W. NLRP3 Inflammasome Involves in the Acute Exacerbation of Patients with Chronic Obstructive Pulmonary Disease. Inflammation. 2018;41(4):1321–33. doi: 10.1007/s10753-018-0780-0 29656319

[38] Pascual-González Y, López-Sánchez M, Dorca J, Santos S. Defining the role of neutrophil-to-lymphocyte ratio in COPD: a systematic literature review. Int J Chron Obstruct Pulmon Dis. 2018;13:3651–62. doi: 10.2147/COPD.S178068 30464448 PMC6225854

