# Supplementary figures and images for: Non-linear association between neutrophil-to-lymphocyte ratio and 90-day mortality in patients with pneumonia receiving glucocorticoids alone or in combination with other immunosuppressants: A retrospective cohort study
Source: PLoS One. 2025 Aug 18;20(8):e0329616. doi: 10.1371/journal.pone.0329616 (PMC12360572; doi:10.1371/journal.pone.0329616)

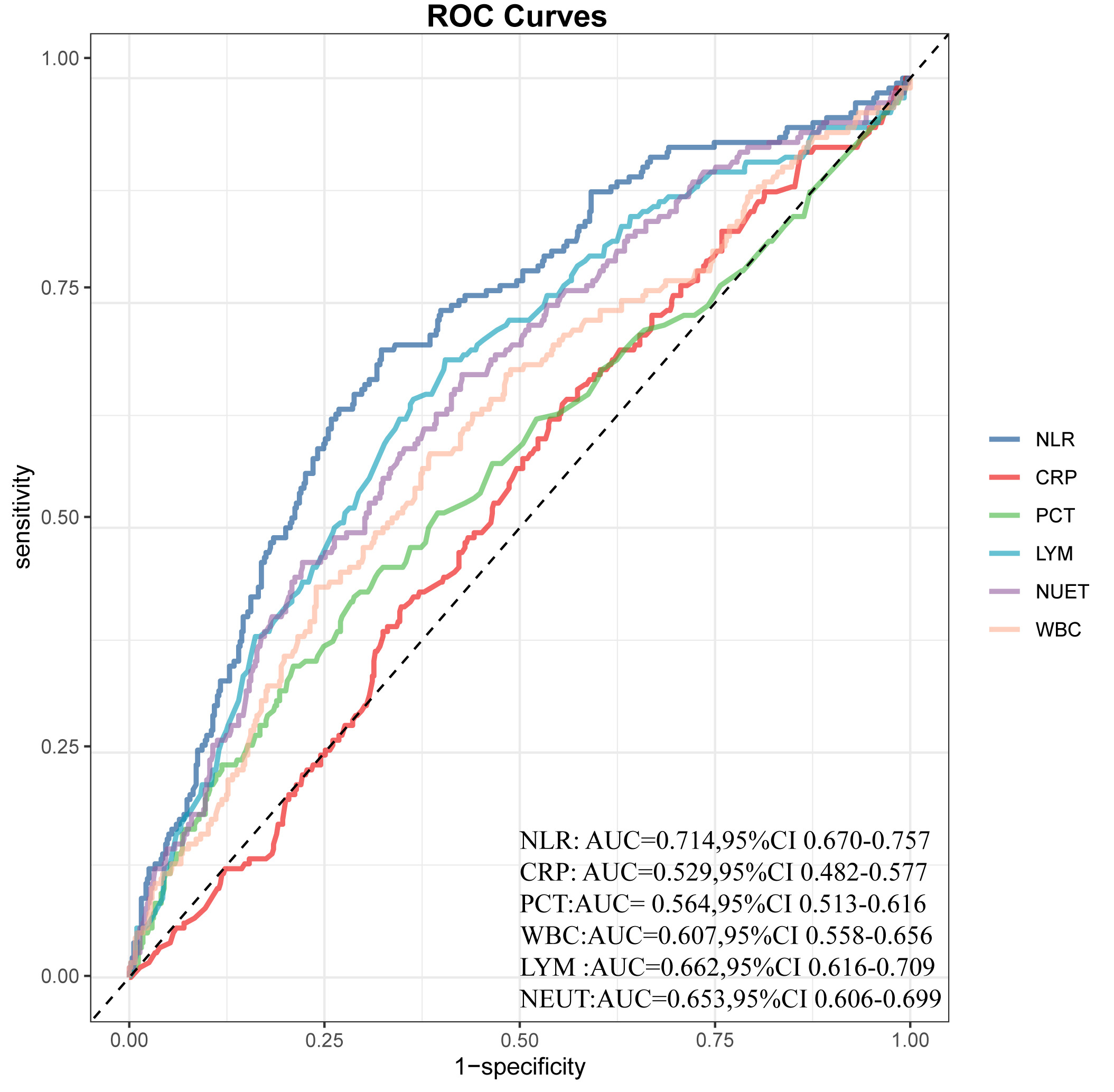

Supplement: S1 Fig — (TIF) [file pone.0329616.s001.tif]
