# Supplementary material for: Non-linear association between neutrophil-to-lymphocyte ratio and 90-day mortality in patients with pneumonia receiving glucocorticoids alone or in combination with other immunosuppressants: A retrospective cohort study
Source: PLoS One. 2025 Aug 18;20(8):e0329616. doi: 10.1371/journal.pone.0329616 (PMC12360572; doi:10.1371/journal.pone.0329616)
Supplement: S2 Table — NLR: Neutrophil/Lymphocyte Ratio; CRP: C-Reactive Protein; PCT: Procalcitonin; WBC: White Blood Cell Count; LYM: Lymphocyte Count; NEUT: Neutrophil Count; NPV: negative predictive value; PPV: positive predictive value; AUC: area-under-curve. (DOCX) [file pone.0329616.s003.docx]

**S2 Table Cut-Off points of NLR,CRP,PCT,WBC,LYM and NEUT for 90-day mortality.**

| Risk factors | Cut-off value | Sensitivity(%) | Specificity(%) | NPV(%) | PPV(%) | AUC | 95% Confidence Interval |
| --- | --- | --- | --- | --- | --- | --- | --- |
| NLR | 9.34 | 69.78 | 67.70 | 86.35 | 43.34 | 0.714 | 0.670-0.757 |
| CRP | 6.21 | 63.74 | 44.55 | 77.63 | 28.93 | 0.529 | 0.482-0.577 |
| PCT | 0.73 | 34.62 | 78.99 | 77.33 | 36.84 | 0.564 | 0.513-0.616 |
| WBC | 8.85 | 58.24 | 61.48 | 80.61 | 34.87 | 0.607 | 0.558-0.656 |
| LYM | 0.82 | 68.68 | 59.53 | 84.30 | 37.54 | 0.662 | 0.616-0.709 |
| NEUT | 6.69 | 67.03 | 57.39 | 83.10 | 35.78 | 0.653 | 0.606-0.699 |

NLR: Neutrophil/Lymphocyte Ratio;CRP:C-Reactive Protein; PCT:Procalcitonin; WBC:White Blood Cell Count;LYM:Lymphocyte Count;NEUT:Neutrophil Count;NPV:negative predictive value; PPV:positive predictive value;AUC: area-under-curve.
