# Supplementary material for: Non-linear association between neutrophil-to-lymphocyte ratio and 90-day mortality in patients with pneumonia receiving glucocorticoids alone or in combination with other immunosuppressants: A retrospective cohort study
Source: PLoS One. 2025 Aug 18;20(8):e0329616. doi: 10.1371/journal.pone.0329616 (PMC12360572; doi:10.1371/journal.pone.0329616)
Supplement: S3 Table — NLR, Neutrophil-to-Lymphocyte Ratio. Model 1: unadjusted. Model 2: adjusted for age, sex. Model 3: adjusted for age, sex, asthma, COPD, CHD, DM and CRF. Model 4 adjust for age, sex, asthma, COPD, CHD, DM, CRF, smoke, alcoholism, PLT, AST, CRE, Na and PSI. (DOCX) [file pone.0329616.s004.docx]

**S3 Table Sensitivity analysis**

| **Variable** | **Model 1** | | **Model 2** | | **Model 3** | | **Model 4** | |
| --- | --- | --- | --- | --- | --- | --- | --- | --- |
|  | **HR (95%CI)** | ***P* value** | **HR (95%CI)** | ***P* value** | **HR (95%CI)** | ***P* value** | **HR (95%CI)** | ***P* value** |
| NLR |  |  |  |  |  |  |  |  |
| Q1(<10) | 1(Ref) |  | 1(Ref) |  | 1(Ref) |  | 1(Ref) |  |
| Q2(≥10) | 3.5 (2.58~4.74) | <0.001 | 3.56 (2.62~4.84) | <0.001 | 3.54 (2.6~4.83) | <0.001 | 3.15 (2.29~4.32) | <0.001 |

NLR,Neutrophil-to-Lymphocyte Ratio.

Model 1:unadjusted.

Model 2:adjusted for age,sex.

Model 3:adjusted for age,sex,asthma,COPD,CHD,DM and CRF.

Model 4 adjust for age, sex, asthma,COPD,CHD,DM,CRF, smoke,alcoholism,PLT,AST,CRE,Na and PSI.
