# Supplementary material for: Non-linear association between neutrophil-to-lymphocyte ratio and 90-day mortality in patients with pneumonia receiving glucocorticoids alone or in combination with other immunosuppressants: A retrospective cohort study
Source: PLoS One. 2025 Aug 18;20(8):e0329616. doi: 10.1371/journal.pone.0329616 (PMC12360572; doi:10.1371/journal.pone.0329616)
Supplement: Attachment — Submitted filename: Major Comment Dr Naif Taleb Ali.pdf [file pone.0329616.s005.pdf]

General Comments:

The manuscript investigates a clinically relevant and timely topic regarding the prognostic value of the neutrophil-to-lymphocyte ratio (NLR) in pneumonia patients treated with glucocorticoids or combined immunosuppressants. The non-linear approach and identification of an inflection point (NLR = 16.475) are novel and insightful. The statistical methodology appears robust, and the findings could be impactful for clinical risk stratification.

However, the manuscript would benefit from several improvements and clarifications as outlined below:

Major Comments:

1. Justification for NLR Threshold:

The inflection point at NLR = 16.475 is statistically derived, but its clinical interpretability needs more discussion. Why this specific value, and how might clinicians use it?

2. Causal Inference Limitations:

As the study is retrospective and observational, emphasize limitations regarding causality and unmeasured confounding.

3. Ethics and Consent Clarification:

The ethics statement says consent was obtained, yet data were also anonymized and from a repository. Clarify how consent was handled, especially for patients in the original Dryad dataset.

4. Clinical Heterogeneity:

The cohort includes a wide age range and varying comorbidities. Did you consider excluding terminally ill or immunocompromised subgroups for sensitivity?

5. Missing Data:

The use of multivariate imputation is good, but detail how much data was missing per variable, and why Bayesian Ridge was preferred.
