# Supplementary material for: Non-linear association between neutrophil-to-lymphocyte ratio and 90-day mortality in patients with pneumonia receiving glucocorticoids alone or in combination with other immunosuppressants: A retrospective cohort study
Source: PLoS One. 2025 Aug 18;20(8):e0329616. doi: 10.1371/journal.pone.0329616 (PMC12360572; doi:10.1371/journal.pone.0329616)
Supplement: Attachment — Submitted filename: Minor Comment Dr Naif Taleb Ali.pdf [file pone.0329616.s006.pdf]

General Comments:

The manuscript investigates a clinically relevant and timely topic regarding the prognostic value of the neutrophil-to-lymphocyte ratio (NLR) in pneumonia patients treated with glucocorticoids or combined immunosuppressants. The non-linear approach and identification of an inflection point (NLR = 16.475) are novel and insightful. The statistical methodology appears robust, and the findings could be impactful for clinical risk stratification.

However, the manuscript would benefit from several improvements and clarifications as outlined below:

Minor Comments:

1. Language and Grammar:

Moderate grammar issues throughout (e.g., article use, run-on sentences). A professional English editing service is recommended.

2. Figures and Legends:

Figure legends should be self-explanatory. Please describe what variables were adjusted for in each survival or forest plot figure.

3. Terminology Consistency:

Maintain consistent terminology for NLR throughout (sometimes written with or without hyphens).

4. Abstract:

Clarify in the abstract that the non-significant association on the right side of the inflection point indicates a plateau in risk.

Recommendation: Major Revision – The study has merit, but methodological clarifications and editorial polishing are required to improve its rigor and readability.
