# Supplementary material for: Non-linear association between neutrophil-to-lymphocyte ratio and 90-day mortality in patients with pneumonia receiving glucocorticoids alone or in combination with other immunosuppressants: A retrospective cohort study
Source: PLoS One. 2025 Aug 18;20(8):e0329616. doi: 10.1371/journal.pone.0329616 (PMC12360572; doi:10.1371/journal.pone.0329616)
Supplement: Attachment — Submitted filename: PONE-D-25-10696.docx [file pone.0329616.s007.docx]

**Non-linear association between Neutrophil-to-Lymphocyte Ratio and 90-day mortality in patients with pneumonia receiving glucocorticoids alone or in combination with other immunosuppressants: a retrospective cohort study**

**1. Originality**

While NLR has been studied in many contexts, the novelty here is slightly limited unless compared head-to-head with other biomarkers in the same population.

**2. Materials and methods**

A. Limited details on how missing data was handled, although Bayesian Ridge imputation is mentioned.
B. No validation cohort was used, which limits generalizability.

3. **Statistically analysis**

A. NLR was treated as both a continuous and categorical variable, but external validation of the threshold (16.475) is missing.
B. No use of ROC curves or AUC to assess predictive performance, which would be useful for clinical implementation.

**4. Results**

A. Results may not be generalizable beyond hospitalized Chinese populations or other types of immunosuppressive treatments.
B. Over interpretation risk: NLR may correlate with illness severity but isn’t necessarily causative.

**5. Recommendations**

• Use a prospective cohort or RCT-based subgroup for stronger causal inference.

• Include external validation cohort to confirm findings.

• Compare NLR’s prognostic value against CRP, procalcitonin, and other inflammatory markers.

• Investigate clinical cutoffs with ROC analysis to enhance practical utility.
