# Supplementary material for: Non-linear association between neutrophil-to-lymphocyte ratio and 90-day mortality in patients with pneumonia receiving glucocorticoids alone or in combination with other immunosuppressants: A retrospective cohort study
Source: PLoS One. 2025 Aug 18;20(8):e0329616. doi: 10.1371/journal.pone.0329616 (PMC12360572; doi:10.1371/journal.pone.0329616)
Supplement: Attachment — Submitted filename: Response to Reviewers.docx [file pone.0329616.s009.docx]

**Revision Rebuttal**

Dear Editor and reviewer:

On behalf of my co-authors, I greatly appreciate the careful review and comments from both you and the reviewers. We believe that by implementing the suggested changes, our manuscript entitled “Non-linear association between neutrophil-to-lymphocyte ratio and 90-day mortality in patients with pneumonia receiving glucocorticoids alone or in combination with other immunosuppressants: a retrospective cohort study”(Number: PONE-D-25-10696) has improved and is more suitable for submission to“PLOS ONE”.

Herein, we have included point-by-point responses for each of the comments in the attached document and have revised our manuscript accordingly. The revised sections were highlighted in YELLOW.

Furthermore, the manuscript has been proofread native English-speaking editor from a professional English language editing company, thus improving the overall language and reliability of the manuscript.

We hope this revised manuscript has addressed your concerns and look forward to your positive responses to the revised manuscript.

The authors declare no conflict of interest regarding this work. All authors have read the revised manuscript and approved it for submission to “PLOS ONE”. Please do not hesitate to contact us if we can be of any further assistance.

Thank you and best regards.

The main corrections in manuscript and the responses to the reviewer's comments are as followings:

**Reviewer #1:**

**Comment 1**

1. Justification and Clinical Implication of the Inflection Point (NLR = 16.475):While the inflection point is statistically supported, more clinical interpretation is needed. How can clinicians utilize this cutoff in practice? Consider discussing its applicability in clinical decision-making.

**Response:**

Thank you for your professional review recommendations;As you mentioned,how the inflection point value of NLR is applied in clinical practice is indeed of crucial importance.Based on your review comments, we have conducted an in-depth discussion on the significance of NLR inflection point values in clinical practice, the revised content of it as follows:“The NLR,derived from routine complete blood counts,serves as an inflammation biomarker reflecting systemic immune dysregulation.Elevated NLR values exhibit significant association with 90-day mortality in patients with pneumonia,necessitating heightened clinical attention to inflammatory status.Its utility as a rapid,cost-effective,and widely accessible biomarker is particularly valuable in severe pneumonia,enabling prompt assessment of inflammatory burden and may provide clinicians with quick stratification.This finding suggests that elevated NLR may portend adverse hospital outcomes, potentially indicative of greater disease severity and suboptimal anti-inflammatory treatment response.Specifically, an NLR threshold ≥16.475 demonstrates significant predictive value for 90-day mortality in this population”.

We have amended this in the manuscript and have highlighted it in yellow.Please see line 234-242,page 12 in the revised manuscript.please review again.

**Comment 2**

2. Handling of Confounding and Causal Inference:

As this is a retrospective observational study, the authors should more explicitly acknowledge and discuss the potential for residual confounding and the limitations in inferring causality, despite adjustment for multiple covariates.

**Response:**

Thank you for your positive advice.We have amended this in the revised manuscript and the revised content of is as follows:“First,due to the nature of the observational study, we only examined the correlation of NLR with 90-day mortality, and could not establish the cause-effect relationship between them.Second,despite performing regression, stratified analysis,there is still a possibility of residual confounding from unmeasured or unknown factors”. Please see line 307-310 page 15 in the revised manuscript.And we have highlighted it in yellow.

**Comment 3**

3. Ethical Considerations and Informed Consent:

The manuscript mentions both anonymized datasets and informed consent. Clarify whether all patients from the Dryad database gave consent, and under what conditions. Transparency is essential in ethics reporting.

**Response:**

Thank you for your valuable suggestion.We have updated the Ethics Statement to clarify that the original study (Li et al. 2020) received ethical approval from China-Japan Friendship Hospital (Approval No. 2015-86) with a waiver of informed consent, as it used only anonymized retrospective data. Our secondary analysis adhered to the same conditions, using fully anonymized data without identifiable information, thus requiring no additional consent. This is explicitly stated in the Materials and Methods section and highlighted it in yellow. Specifically as follows:“Informed consent was waived due to the retrospective nature of the study”.Please see line 74 page 4.

Exclusion criterion (2) was revised to improve precision.Please see page 5,line 96.

**Comment 4**

4. Subgroup and Sensitivity Analyses:

Subgroup results suggest effect modification by age and smoking. However, sensitivity analyses could be strengthened by examining other clinically relevant subgroups or using alternative cutoffs for NLR.

**Response:**

We are grateful for your insightful suggestions.In the forest plot for age groups, the OR values of both groups are in the same direction. In the smoking variable group, the small number of current smokers in the third group may have led to unstable results.

And according to your advice,we conducted a sensitivity analysis using alternative NLR cut off value 10.0, validating the outcome data,Specifically as follows:

**(1) Statistical analysis section:**“To assess the robustness of our findings,sensitivity analysis was conducted by reclassifying NLR according to the established optimal cutoff value of 10.0,derived from previous studies[17,32]”.Please see page 6-7,lines 141-143 and highlighted it in yellow.

**(2) Result section:**“**Sensitivity analysis**

For sensitivity analysis,the NLR was dichotomised using the optimal cutoff value of 10.0,previously established via ROC curve analysis,confirming a persistent association between NLR and 90-day mortality in patients with pneumonia (HR: 3.15, 95% CI 2.29-4.32, *p*<0.001), as detailed in S3 Table”.Please see page 11,lines 223-226.

**(3)Discussion section:**“Both the sensitivity and stratified analyses confirmed that the association between NLR and 90-day mortality remained robust”.Please see page 12,lines 242-243 and highlighted it in yellow.

**S3 Table Sensitivity analysis**

| **Variable** | **Model 1** | | **Model 2** | | **Model 3** | | **Model 4** | |
| --- | --- | --- | --- | --- | --- | --- | --- | --- |
|  | **HR (95%CI)** | ***P* value** | **HR (95%CI)** | ***P* value** | **HR (95%CI)** | ***P* value** | **HR (95%CI)** | ***P* value** |
| NLR |  |  |  |  |  |  |  |  |
| Q1(<10) | 1(Ref) |  | 1(Ref) |  | 1(Ref) |  | 1(Ref) |  |
| Q2(≥10) | 3.5 (2.58~4.74) | <0.001 | 3.56 (2.62~4.84) | <0.001 | 3.54 (2.6~4.83) | <0.001 | 3.15 (2.29~4.32) | <0.001 |

NLR,Neutrophil-to-Lymphocyte Ratio.

Model 1:unadjusted.

Model 2:adjusted for age,sex.

Model 3:adjusted for age,sex,asthma,COPD,CHD,DM and CRF.

Model 4 adjust for age, sex, asthma,COPD,CHD,DM,CRF, smoke,alcoholism,PLT,AST,CRE,Na and PSI.

**Comment 5**

5. Missing Data Handling:

The authors applied multivariate imputation using a Bayesian Ridge estimator. Please detail the percentage of missing data per variable and justify why this imputation method was selected over others.

**Response:**

**Thank you for your comments.**

We have supplemented the list of missing values. The details of the missing value are shown in Supplementary S1 Table .Supplements were made in the revised manuscript. The results are as follows:

**S1 Table Details of missing values.**

| **Variables** | **The number of missing values** | **The percent of**  **missing values (%)** |
| --- | --- | --- |
| Age | 0 | 0 |
| Sex, n (%) | 0 | 0 |
| Asthma, n (%) | 0 | 0 |
| COPD, n (%) | 0 | 0 |
| CHD, n (%) | 0 | 0 |
| DM, n (%) | 0 | 0 |
| CRF, n (%) | 0 | 0 |
| Smoke, n (%) | 0 | 0 |
| Alcoholism, n (%) | 0 | 0 |
| PLT×109/L | 11 | 1.5805 |
| AST(U/L) | 11 | 1.5805 |
| CRE(μmmol/L) | 9 | 1.2931 |
| Na(mmol/L) | 14 | 2.0115 |
| PSI | 0 | 0 |

Abbreviations:COPD,chronic obstructive pulmonary disease;CHD,coronary heart disease; DM, diabetes mellitus;CRF,coronary renal failure;PLT,platelet;AST,Aspartate Aminotransferase; CRE, creatinine;PSI,pneumonia severity index (PSI) score

Our study conducted multivariate imputation using a Bayesian Ridge estimator.Its main virtues are that imputations are restricted to the observed values and that it can preserve non-linear relations even if the structural part of the imputation model is wrong. Finally, the function mice.impute.sample just takes a random draw from the observed data, and imputes these into missing cells. This function does not condition on any other variable.The idea of MI is to take into account uncertainty in predicting missing values by creating multiple complete datasets.

Multiple imputation (MI) is an advanced method in handling missing values. In contrast to single imputation, MI creates a number of datasets (denoted by m) by imputing missing values.That is, one missing value in original dataset is replaced by m plausible imputed values.These values take imputation uncertainty into consideration.

We have revised the expression regarding the handling of missing values in the statistical analysis section to be more precise and highlighted it in yellow. Please refer to page 7, lines 143-146 in the revised manuscript.

**Minor Comments:**

**Comment 1**

1. Language and Grammar:

The manuscript contains several grammatical errors and awkward phrasing. Consider professional language editing to improve readability and clarity.

**Response:**

Thank you for your positive advice.We have corrected the grammar mistakes and inappropriate expressions.The English content in the paper has been professionally edited and checked by native English speakers.Please review again.

**Comment 2**

2. Terminology Consistency:

Ensure consistent use of terms, especially for "neutrophil-to-lymphocyte ratio (NLR)", which is sometimes written with and without hyphens or inconsistent abbreviations.

**Response:**

Thank you for your valuable suggestion.In accordance with your recommendation,we have thoroughly rechecked the the consistent use of terms,especially for "neutrophil-to-lymphocyte ratio (NLR)".And we have made the necessary corrections to ensure consistency throughout the manuscript.Please review again.

**Comment 3**

3. Figure Legends and Interpretation:

Legends should clearly indicate all adjustments made in regression or Kaplan-Meier curves. Currently, figures lack self-contained explanations.

**Response:**

Thank you for remark.Following your advice,I have supplemented the legend of the Kaplan-Meier curve.The revised content is as follows:“Adjusted for age, sex, asthma,COPD,CHD,DM,CRF,smoke,alcoholism,PLT,AST,CRE,Na and PSI.” Specifically,it is on page 10,line 206,and highlighted it in yellow.

**Comment 4**

4. Abstract Refinement:

The abstract should explicitly state that the mortality risk plateaued beyond the NLR threshold, to aid understanding for readers without statistical expertise.

**Response:**

Thank you for your valuable suggestion to improve the quality of our manuscript.Based on your proposal,we have added it in the abstract of revised manuscript as“after exceeding the threshold, the risk of 90-day mortality tended to stabilize”.Specifically,it is on page 2,lines 35,and highlighted it in yellow.

**Reviewer #2:**

**Comment 1**

1. Originality

While NLR has been studied in many contexts, the novelty here is slightly limited unless compared head-to-head with other biomarkers in the same population.

**Response:**

Thank you for your constructive suggestion.According to your advice,we further explored the prognostic value of NLR in pneumonia patients.Receiver operator characteristic (ROC) curves constructed for NLR and other existing biomarkers are presented in S1 Fig.

Modifications and supplements have been made to the Abstract, Statistical Methods, Results, and Discussion sections of the revised manuscript.

Specifically:

①**Abstract:** The supplementary contents are as follows:

**Methods section in abstract:**“Receiver-operating characteristic curves (ROC) and area under the curves (AUC) were used to assess the ability of different biomarkers to predict the 90-day mortality in patients with pneumonia.”(page 1, lines 21-22) .

**Results section in abstract:**“Furthermore, the ROC curve analysis revealed that NLR was the optimal biomarker for predicting the 90-day mortality with an AUC of 0.714 (95% CI:0.670-0.757).Using 9.34 as the cutoff value of NLR, the sensitivity was 69.8%, and the specificity was 67.7%.”(page 2, lines 30-32).

②**Statistical Methods:**The supplementary contents are as follows:“The Youden index was applied to determine the optimal cut-off value for each variable,and receiver operating characteristic (ROC) curve analysis assessed the sensitivity and specificity of different biomarkers predicting adverse prognosis.The area under the curve (AUC) was used to evaluate prognostic accuracy,with comparisons conducted via a nonparametric approach.Higher AUC values indicate greater discriminatory ability.”(page 6 and lines 137-141).

③**Results:** The supplementary contents are as follows:“**Diagnostic efficiency of NLR compared with other biomarkers**

We further assessed the prognostic value of NLR in patients with pneumonia.ROC curves generated for NLR and established biomarkers were illustrated in S1 Fig.The AUC value for NLR was 0.714.For predicting 90-day mortality,NLR demonstrated superior performance relative to established biomarkers, including WBC,NEUT,LYM, CRP, PCT(AUC_WBC_=0.607,AUC_NEUT_=0.653, AUC_LYM_=0.662, AUC_CRP_=0.529, AUC_PCT_=0.564, *p*<0.05).Using 9.34 as the optimal cutoff value of NLR, the sensitivity was 69.78%, and the specificity was 67.70%(S2 Table).”(page 11, lines 216-222).

S1 figure as follows:

④**Discussion:** The supplementary contents are as follows:“Since the association between NLR and mortality in pneumonia patients has been extensively studied[17-18,32],we compared the predictive value of NLR with currently biomarkers in predicting 90-day mortality in pneumonia patients.We found that NLR performed better than WBC,NEUT,LYM,CRP and PCT.”(page 14, lines 286-289).

**Comment 2**

2. Materials and methods

A. Limited details on how missing data was handled, although Bayesian Ridge imputation is mentioned.

B. No validation cohort was used, which limits generalizability.

**Response :**

A. Thank you for your valuable advice.We have taken your feedback into account and have revised the description in the revised manuscript.The revised contents are as follows:“We used multiple imputations, based on 5 replications and a chained equation approach method in the R MI procedure, to maximize statistical power and minimize bias that might occur to account for missing data[25].The details of the missing value are shown in S1 Table.”(page 7,line 143-146).

B.Thank you for pointing this out.External validation is explained in the limitations of the article, specially as follows:“In the future,prospective cohort studied or subgroups based on randomized controlled trials will be conducted to strengthen causal inference,including the use of external validate cohort to confirm our findings.”Please see page 15, lines 320-322, and which is also a direction for our future research.

**Comment 3**

3. Statistically analysis

A. NLR was treated as both a continuous and categorical variable, but external validation of the threshold (16.475) is missing.

B. No use of ROC curves or AUC to assess predictive performance, which would be useful for clinical implementation.

**Response :**

A.Thank you for your comment.We appreciate your professional advice,and We have made supplements in the limitations, and the specific content is as follows:“and external validation of the 16.475 threshold will be performed in subsequent studies”.Please see page 15, lines 317-318, and this will be a direction of our research and we will take this as our next task.

B.To test the predictive performance, we added the ROC curve and the area under the curve,as detailed S1 Fig.

Modifications and supplements have been made to the Abstract, Statistical Methods, Results, and Discussion sections of the manuscript. Specifically:

**①Abstract**: The supplementary contents are as follows:

**Methods section in abstract:**“Receiver-operating characteristic curves (ROC) and area under the curves (AUC) were used to assess the ability of different biomarkers to predict the 90-day mortality in patients with pneumonia.”(page 1, lines 21-22) .

**Results section in abstract:**“Furthermore, the ROC curve analysis revealed that NLR was the optimal biomarker for predicting the 90-day mortality with an AUC of 0.714 (95% CI:0.670-0.757).Using 9.34 as the cutoff value of NLR, the sensitivity was 69.8%, and the specificity was 67.7%.”(page 2, lines 30-32).

**②Statistical Methods:** The supplementary contents are as follows:“The Youden index was applied to determine the optimal cut-off value for each variable,and receiver operating characteristic (ROC) curve analysis assessed the sensitivity and specificity of different biomarkers predicting adverse prognosis.The area under the curve (AUC) was used to evaluate prognostic accuracy,with comparisons conducted via a nonparametric approach.Higher AUC values indicate greater discriminatory ability.”(page 6 and lines 137-141).

**③Results:** The supplementary contents are as follows:“**Diagnostic efficiency of NLR compared with other biomarkers**

We further assessed the prognostic value of NLR in patients with pneumonia.ROC curves generated for NLR and established biomarkers were illustrated in S1 Fig.The AUC value for NLR was 0.714.For predicting 90-day mortality,NLR demonstrated superior performance relative to established biomarkers, including WBC,NEUT,LYM, CRP, PCT(AUC_WBC_=0.607,AUC_NEUT_=0.653, AUC_LYM_=0.662, AUC_CRP_=0.529, AUC_PCT_=0.564, *p*<0.05).Using 9.34 as the optimal cutoff value of NLR, the sensitivity was 69.78%, and the specificity was 67.70%(S2 Table).”(page 11, lines 216-222).

S1 figure as follows:

**④Discussion:** The supplementary contents are as follows:“Since the association between NLR and mortality in pneumonia patients has been extensively studied[17-18,32],we compared the predictive value of NLR with currently biomarkers in predicting 90-day mortality in pneumonia patients.We found that NLR performed better than WBC,NEUT,LYM,CRP and PCT.”(page 14, lines 286-289).

**Comment 4**

4. Results

A. Results may not be generalizable beyond hospitalized Chinese populations or other types of immunosuppressive treatments.

B. Over interpretation risk: NLR may correlate with illness severity but isn’t necessarily causative.

**Response :**

A.Thank you for your valuable remark.This point has been explicitly addressed in the limitations section of our manuscript,specially as follows:“Moreover,while our findings are primarily confined to hospitalized pneumonia patients undergoing glucocorticoid-based regimens,the identified inflection point may possess broader applicability to the general population of hospitalized pneumonia patients and external validation of the 16.475 threshold will be performed in subsequent studies.”Specific details can be found on page 15, lines 314-318.

B.Thank you for you comment.According to your suggestion,considering that "the risk" might over-interpret risks, we have deleted "the risk" from the paper, indicated by a strix line, and marked it in red. For details, please refer to page 2, line 35,and page 9, line 170 and page 9,line 175,and page 12,line 231,and page 15,line 325.

**Comment 5**

5. Recommendations

• Use a prospective cohort or RCT-based subgroup for stronger causal inference.

• Include external validation cohort to confirm findings.

•Compare NLR’s prognostic value against CRP, procalcitonin, and other inflammatory markers.

• Investigate clinical cutoffs with ROC analysis to enhance practical utility.

**Response :**

A and B:Thank you for your valuable advice.We appreciate your professional advice.According to your advice,We have made changes to the discussion section as follows“In the future,prospective cohort studied or subgroups based on randomized controlled trials will be conducted to strengthen causal inference,including the use of external validate cohort to confirm our findings.”(page 15,lines 320-322).

C.Thank you for your valuable suggestion.To test the predictive performance, we added the ROC curve and the area under the curve,as detailed S1 Fig.

Modifications and supplements have been made to the Abstract, Statistical Methods, Results, and Discussion sections of the manuscript. Specifically:

①**Abstract:** The supplementary contents are as follows:

**Methods section in abstract:**“Receiver-operating characteristic curves (ROC) and area under the curves (AUC) were used to assess the ability of different biomarkers to predict the 90-day mortality in patients with pneumonia.”(page 1, lines 21-22) .

**Results section in abstract:**“Furthermore, the ROC curve analysis revealed that NLR was the optimal biomarker for predicting the 90-day mortality with an AUC of 0.714 (95% CI:0.670-0.757).Using 9.34 as the cutoff value of NLR, the sensitivity was 69.8%, and the specificity was 67.7%.”(page 2, lines 30-32).

②**Statistical Methods:**The supplementary contents are as follows:“The Youden index was applied to determine the optimal cut-off value for each variable,and receiver operating characteristic (ROC) curve analysis assessed the sensitivity and specificity of different biomarkers predicting adverse prognosis.The area under the curve (AUC) was used to evaluate prognostic accuracy,with comparisons conducted via a nonparametric approach.Higher AUC values indicate greater discriminatory ability.”(page 6 and lines 137-141).

③**Results:** The supplementary contents are as follows:“**Diagnostic efficiency of NLR compared with other biomarkers**

We further assessed the prognostic value of NLR in patients with pneumonia.ROC curves generated for NLR and established biomarkers were illustrated in S1 Fig.The AUC value for NLR was 0.714.For predicting 90-day mortality,NLR demonstrated superior performance relative to established biomarkers, including WBC,NEUT,LYM, CRP, PCT(AUC_WBC_=0.607,AUC_NEUT_=0.653, AUC_LYM_=0.662, AUC_CRP_=0.529, AUC_PCT_=0.564, *p*<0.05).Using 9.34 as the optimal cutoff value of NLR, the sensitivity was 69.78%, and the specificity was 67.70%(S2 Table).”(page 11, lines 216-222).

S1 figure as follows:

④**Discussion:** The supplementary contents are as follows:“Since the association between NLR and mortality in pneumonia patients has been extensively studied[17-18,32],we compared the predictive value of NLR with currently biomarkers in predicting 90-day mortality in pneumonia patients.We found that NLR performed better than WBC,NEUT,LYM,CRP and PCT.”(page 14, lines 286-289).

D. Thank you for your reviewer.We supplemented the optimal cutoff value of NLR,Please refer to S2 Table for details.Specifically as follows:

**S2 Table Cut-Off points of NLR,CRP,PCT,WBC,LYM and NEUT for 90-day mortality.**

| Risk factors | Cut-off value | Sensitivity(%) | Specificity(%) | NPV(%) | PPV(%) | AUC | 95% Confidence Interval |
| --- | --- | --- | --- | --- | --- | --- | --- |
| NLR | 9.34 | 69.78 | 67.70 | 86.35 | 43.34 | 0.714 | 0.670-0.757 |
| CRP | 6.21 | 63.74 | 44.55 | 77.63 | 28.93 | 0.529 | 0.482-0.577 |
| PCT | 0.73 | 34.62 | 78.99 | 77.33 | 36.84 | 0.564 | 0.513-0.616 |
| WBC | 8.85 | 58.24 | 61.48 | 80.61 | 34.87 | 0.607 | 0.558-0.656 |
| LYM | 0.82 | 68.68 | 59.53 | 84.30 | 37.54 | 0.662 | 0.616-0.709 |
| NEUT | 6.69 | 67.03 | 57.39 | 83.10 | 35.78 | 0.653 | 0.606-0.699 |

NLR: Neutrophil/Lymphocyte Ratio;CRP:C-Reactive Protein; PCT:Procalcitonin; WBC:White Blood Cell Count;LYM:Lymphocyte Count;NEUT:Neutrophil Count;NPV:negative predictive value; PPV:positive predictive value;AUC: area-under-curve..
